# Fatigue and quality of life during neoadjuvant chemotherapy of early breast cancer: a prospective multicenter cohort study

**DOI:** 10.1007/s12282-023-01520-y

**Published:** 2023-11-15

**Authors:** Florian Pelzer, Wilfried Tröger, Marcus Reif, Susanne Schönberg, David D. Martin, Cornelia Müller, Isabell Utz-Billing, Thorsten Kühn, Stephan Baumgartner, Marion Kiechle, Daniela Paepke

**Affiliations:** 1 Institute for Integrative Medicine, Witten/Herdecke University, Witten, Germany; 2 Society for Cancer Research, Arlesheim, Switzerland; 3 Gesellschaft Für Klinische Forschung e.V., Berlin, Germany; 4 Tübingen University Children’s Hospital, Tübingen, Germany; 5 Brandenburgisches Brustzentum, Universitätsklinikum Brandenburg an der Havel, Brandenburg an der Havel, Germany; 6 Brustzentrum, Park-Klinik Weissensee, Berlin, Germany; 7 Klinik für Frauenheilkunde und Geburtshilfe, Klinikum Esslingen, Esslingen, Germany; 8 Filderklinik, Filderstadt, Germany; 9 Klinik für Frauenheilkunde und Geburtshilfe, Universitätsklinikum Ulm, Ulm, Germany; 10 Department of Obstetrics and Gynecology, Klinikum Rechts der Isar, Technical University of Munich, Munich, Germany; 11 Spital Zollikerberg, Zurich, Switzerland

**Keywords:** Quality of life, Breast cancer, Neoadjuvant chemotherapy, Cancer-related fatigue, Cohort study

## Abstract

**Background:**

Few measurements of fatigue and quality of life have been performed during neoadjuvant chemotherapy of early breast cancer. This study evaluates fatigue and quality of life experienced by early breast cancer patients during neoadjuvant chemotherapy and their association with different clinical parameters.

**Methods:**

Fifty-four stage I–III patients’ responses to the Multidimensional Fatigue Inventory (MFI) and to the Functional Assessment of Cancer Therapy-Breast (FACT-B) were analyzed by a linear covariance pattern model. Chemotherapy regimen, age, baseline fatigue level, body-mass-index and cancer stage were added to the model to estimate their impact on both outcomes.

**Results:**

All fatigue dimensions worsened in clinically relevant levels. Physical fatigue worsened the most, mental fatigue the least. For quality of life, physical and functional well-being worsened the most. Only emotional well-being improved during chemotherapy. Physical well-being worsened more during standard than during dose-dense chemotherapy, and more during anthracycline than during taxane cycles. Age, body-mass-index and cancer stage had no impact. The higher the fatigue levels at baseline, the less they worsened during chemotherapy.

**Conclusions:**

Further actions to reduce fatigue and improve quality of life during neoadjuvant chemotherapy of early breast cancer are needed. Focus should be laid on the physical dimension. Future research should also investigate the impact of different chemotherapy sequences and densities on fatigue and quality of life.

**Study registration:**

The study was registered in the German Clinical Trials Register in May 2019 (DRKS00016761).

**Supplementary Information:**

The online version contains supplementary material available at 10.1007/s12282-023-01520-y.

## Introduction

Neoadjuvant chemotherapy of breast cancer is the administration of chemotherapy prior to definitive breast surgery [1]. The proportion of early breast cancer patients treated with neoadjuvant chemotherapy increased in Germany from 6% in 2008 to 18% in 2018, resulting in 58% of all chemotherapies for early breast cancer being neoadjuvant [2, 3]. Clinical evidence has shown that neoadjuvant chemotherapy can lead to surgical downstaging of the tumor, render inoperable tumors resectable, de-escalate axillary surgery in patients with clinically positive nodes and provide prognostic information regarding risk of recurrence and prognosis of adjuvant treatment, i.e., after surgery [1]. Therefore, early breast cancer patients with either high-risk hormone-receptor positive, human epidermal growth factor receptor 2 (HER2) positive, triple-negative, inflammatory or locally advanced breast cancer are eligible for neoadjuvant chemotherapy [4]. Neoadjuvant chemotherapy of early breast cancer usually lasts 18–24 weeks and includes anthracycline, cyclophosphamide and taxane cycles together with, if indicated, carboplatin or monoclonal antibodies [5].

Fatigue levels have been shown to be 30% higher in breast cancer patients than in the general population [6]. Fatigue during cancer has been defined as cancer-related fatigue (CRF), “a distressing, persistent, subjective sense of physical, emotional, and/or cognitive tiredness or exhaustion related to cancer or cancer treatment that is not proportional to recent physical activity and that interferes with usual functioning” [7]. CRF levels have been associated with different demographic, clinical and therapeutic parameters. Female patients aged 18–40 have shown a stronger increase in fatigue after cancer diagnosis than those aged 61–70 [6]. CRF levels have been significantly higher in tumor stage IV than in stages I–III [6]. In the adjuvant setting, biweekly cycles of anthracyclines and taxanes have generated higher CRF levels than the same combination given on a three-weekly basis [8]. The sequence, however, of anthracyclines and taxanes given as adjuvant chemotherapy has had no impact on quality of life according to one study [9]. In addition, a high body-mass-index (BMI) at the beginning of neoadjuvant chemotherapy has not been a predictor for CRF increase during the treatment [10].

Chemotherapy affects physical dimensions of CRF and health-related quality of life (HRQoL) more than other dimensions. Physical dimensions of CRF and HRQoL have worsened during adjuvant and neoadjuvant chemotherapy in different studies of early breast cancer patients [11–13]. Other dimensions did not change (e.g., mental fatigue and motivation [14]) or even improved (emotional HRQoL [12]).

Few and relatively small studies on CRF and HRQoL dimensions have been performed with early breast cancer patients during neoadjuvant chemotherapy [12, 13, 15, 16], which is why further evaluation of CRF and HRQoL in this population is needed. Their development might differ from that of the adjuvant setting, as surgery has been shown to worsen CRF and HRQoL [17] and thereby alter the patient’s perception of both endpoints during chemotherapy [11]. The present cohort study therefore aimed at generating more data to identify clinical needs. In addition, to our knowledge, no study has evaluated the effect of age, BMI, cancer stage and different chemotherapy regimens on CRF and HRQoL of early breast cancer patients during neoadjuvant chemotherapy. The aims of the present study are therefore, first, to evaluate the levels of CRF and HRQoL experienced by early breast cancer patients during neoadjuvant chemotherapy and, second, to evaluate their association with different clinical parameters.

## Patients and methods

### Participants and study design

This is a prospective, multicenter cohort study. None of the consulted ethics committees raised concerns regarding its conduct, and the study was registered in the German Clinical Trials Register on 6 May 2019 (DRKS00016761). Participants in this study were female patients with histologically confirmed early breast cancer, i.e., Union for International Cancer Control (UICC) stages I–III, excluding patients with no tumor (T_0_), with a carcinoma in situ (T_is_) or with a microinvasion (T_1mic_), and scheduled to receive neoadjuvant chemotherapy according to guidelines [4]. Participants had an Eastern Cooperative Oncology Group (ECOG) performance score of 0–2, an age between 18 and 80 years, no current pregnancy, no concomitant medication critically influencing their ability to follow the requirements of the clinical observation, no participation in a clinical trial or use of an investigational agent from four weeks before the first until the last chemotherapy cycle, and had given signed informed consent. Participants were treated in four certified breast cancer centers, two in Southern and Eastern Germany each, in one large- and one medium-sized city in each region (Munich, Berlin, Esslingen, Brandenburg a. d. Havel).

The observation started with participant inclusion, before the first cycle of neoadjuvant chemotherapy, and ended 2 weeks after surgery or, if surgery was not performed, 2 weeks after the last chemotherapy cycle, in order to measure the effect of the last treatment step after a time interval comparable to the previous steps. In addition to data from patient records, standardized questionnaires were filled out by the patients.

All collected data were transferred into the clinical data management system Marvin EDC (XClinical GmbH, Munich, Germany) using electronic case report forms (eCRFs) developed by the Gesellschaft für klinische Forschung e.V. (GKF). Permanent centralized monitoring was performed together with on-site-monitoring in each center 8 weeks after inclusion of the first participant and thereafter on a risk-based approach to ensure patient safety, data completeness and accuracy. Data managers of the GKF checked the eCRF database for new entries on a daily basis. Conspicuous data (e.g., missing data, data outside reference ranges) were flagged as query within the eCRF system, eliciting a request for check by the data-entry study personnel. A patient's eCRF documentation could not be electronically signed and locked if queries were still unresolved. At the end of documentation and data cleaning the data were transferred into SAS data formats and analyzed with SAS for Windows version 9.4 (SAS Institute Inc., Cary, NC, USA).

### Demography, tumor factors and chemotherapy regimens

Demographic and tumor factors were collected at the qualifying examination including age, menopausal status, tumor stage, nodal stage, tumor grading, Ki-67 score, estrogen-receptor (ER) status, progesterone-receptor (PR) status, human epidermal growth factor receptor-2 (HER-2) status, ECOG-score, BMI and concomitant diseases.

Recording of chemotherapy drugs and regimens started with the first chemotherapy cycle, which defined the baseline visit. Thereafter, visits during the whole observation time were documented every 2 or 3 weeks, depending on the density of the anthracycline cycle, i.e., whether it was given every 2 (dose-dense) or 3 weeks (standard).

### Patient-reported quality of life and symptoms assessment

Primary outcomes of this study were ‘general fatigue’, as assessed by the Multidimensional Fatigue Inventory (MFI), and HRQoL as assessed by the Trial Outcome Index (TOI) of the Functional Assessment of Cancer Therapies – Breast Cancer (FACT-B). The MFI is a self-report instrument designed to measure fatigue. It consists of 20 items grouped in five dimensions: general fatigue, physical fatigue, mental fatigue, reduced motivation and reduced activity. Patients indicate on a 1- to 5-point scale to what extent the corresponding statement applies to them. One score between 4 and 20 is generated per dimension. The higher the score, the higher the fatigue perceived by the patient [18]. The FACT-B is a self-report instrument designed to measure multidimensional HRQoL in patients with breast cancer. In its fourth version, it consists of 37 items with five ordered categorical answers. FACT-B items are grouped into the subscales physical, social, emotional and functional well-being, with score ranges of 0–28, 28, 24, 28, respectively, and an additional breast cancer subscale with a score range of 0–40. The TOI summarizes the physical, the functional well-being and the breast cancer subscales with a score range of 0 to 96. The FACT-B total score summarizes all subscales with a score range of 0 to 148. The higher the score, the better is the HRQoL [19]. The MFI and FACT-B were completed by participants before chemotherapy at visits defined by the protocol, i.e., every 2 or 3 weeks (see above).

Secondary outcomes of this study were the incidence and severity of adverse events arising during neoadjuvant chemotherapy. The severity of adverse events was documented according to the Common Terminology Criteria for Adverse Events (CTCAE), version 5.0. Adverse events were classified according to the Medical Dictionary for Regulatory Activities (MedDRA), version 22.1, by data managers of the GKF.

### Statistical analyses

While no confirmatory interpretation of statistical results was intended, the planned sample size of 50 patients was chosen for having a power of 80% to detect a standardized mean difference of 0.4 for the change from baseline to the end of the study in one of the primary efficacy parameters. All analyses of the primary and secondary outcomes were done with the Full Analysis Set which included all patients fulfilling the inclusion/exclusion criteria, having given informed consent and having at least one follow-up after the baseline visit. There was only one patient who dropped out of the study immediately after the qualifying examination and for whom no further efficacy- or safety-relevant data were documented.

Demographic and baseline characteristics, as well as outcome parameters, are presented by descriptive statistics, including median and interquartile range (IQR) in case of quantitative data, and contingency tables showing absolute and relative frequencies for categorical data. Results of statistical tests are shown as estimates, 95% confidence intervals and corresponding *p*-values, which were interpreted in exploratory intent only, which is why no adjustment for multiple testing was done. As an exception, in accordance with good statistical practice, the *p* values of the two primary outcome parameters were evaluated after Bonferroni-Holm adjustment for multiple testing, with statistical significance claimed only when the smaller *p* value was less than half the global α error level, i.e., 2.5%.

Missing data were considered in general as 'missing at random', which is reasonable since only five out of 54 included patients (9.3%) terminated chemotherapy early, one directly lost to follow-up after qualifying examination, the other four after 14 to 21 weeks of chemotherapy. Missing data were not substituted, with two exceptions. First, missing individual items of the MFI and FACT-B questionnaires were supplemented according to the respective guidelines. Second, MFI and FACT-B baseline scores still missing after this supplementing procedure were estimated by linear regression based on all available data, with patients and patient*time interactions included as fixed factors in the model.

The MFI and FACT-B results of all Full Analysis Set patients were analyzed by a linear covariance pattern model which included the respective baseline values and baseline*time interactions as independent covariates. In addition, the model contained the density of anthracycline cycles (standard vs. dose-dense), the time since start of chemotherapy, and their interaction as fixed factor and covariate, respectively. Dependent parameters were the change from baseline of the specific dimensions of the MFI or FACT-B, respectively. Of these, the MFI general fatigue scale and the FACT-B TOI represented the primary efficacy parameters of the study, while other MFI dimensions and FACT-B subscales were regarded as the secondary parameters. To account for dependencies between intra-individual measurements, a first-order autoregressive covariance structure between adjacent time points was chosen. Since most chemotherapy regimens consisted of two drug combinations, epirubicin with or without cyclophosphamide (Epi ± Cyc) and (nab-)paclitaxel with or without carboplatin (Pac ± Car), administered in this or reverse order, in a subsequent analysis of these regimens we additionally included the type and order of these drugs in the statistical model together with their respective interactions with the duration of their administration. Density of chemotherapy cycles was removed from this model, since this factor was found to be uninformative in this parameter constellation.

In sensitivity analyses of the primary statistical model, possible confounders (age, BMI, UICC stage) were added to the original model in a multivariate variable selection procedure to estimate their impact on primary outcome parameters.

Analysis of adverse events focused on patients receiving Epi ± Cyc and Pac ± Car due to the small number of patients (*N* = 3) and adverse events under other drug combinations. Adverse events were evaluated according to their MedDRA classification, severity and prevalence during treatment. Adverse events were also classified into “new onset” and “ongoing” adverse events and were analyzed separately. “New onset” describes adverse events documented for the first time in the patient’s observation time. “Ongoing” adverse events have already been documented for this patient during the previous drug combination. Incidences of AEs of severity grade ≥ 2 between the drug combinations were estimated separately for each diagnose using exact logistic regression. No order or interaction term was considered in this specific model for the sake of model stability. Yet, for 'new onset' adverse events, the model additionally included the individual patients as strata to account for within-patient dependencies. Over all AEs, hazard rates (HR; number of adverse events per week per patient) were calculated using a Poisson regression model including the drug combination as fixed factor and the (logarithmized) duration of the respective combination’s treatment time as offset parameter to account for different drug treatment durations. For 'new onset' adverse events, the order of the respective drug combination and order*drug combination interaction were also added to the model, as well as a random factor controlling for the dependencies between intra-individual measurements of consecutive sequences. Finally, reasons for premature chemotherapy termination were listed in the supplementary data.

## Results

### Patient characteristics

From June 2019 to April 2020, 54 patients from four German centers were included in the cohort and were followed up until February 2021. Patient characteristics are summarized in Table 1. The median age at baseline was 50 (IQR 43–62). Fifty-one (94%) and three (6%) patients had an ECOG-score of 0 and 1, respectively, none had a higher score. The median BMI was 24 (IQR 22–28). The median MFI general fatigue and FACT-B TOI scores at baseline were 9 (IQR 6–11) and 71 (IQR 63–81), respectively.

**Table 1:** Demographic data of the total patient cohort and of the subcohorts receiving standard or dose-dense neoadjuvant chemotherapy

	Total (*N* = 54)	Standard (*N* = 25)	Dose-dense (*N* = 29)	*P* value
Age (years)	50 (43–62)	57 (46–68)	49 (40–59)	0.062
Menopausal status				
Premenopausal	22 (41%)	5 (20%)	17 (59%)	0.013
Perimenopausal	4 (7%)	3 (12%)	1 (3%)	
Postmenopausal	27 (50%)	16 (64%)	11 (38%)	
Missing	1 (2%)	1 (4%)	0	
Histologically assessed tumor stage				
cT1 (cT1_mic_ excl.)	15 (28%)	8 (32%)	7 (25%)	0.988
cT2	32 (59%)	13 (52%)	19 (66%)	
cT3	4 (7%)	3 (12%)	1 (3%)	
cT4	2 (4%)	1 (4%)	1 (3%)	
cTX	1 (2%)	0	1 (3%)	
Nodal stage				
cN0	29 (54%)	15 (60%)	14 (48%)	0.862
cN1	20 (37%)	9 (36%)	11 (38%)	
cN2	4 (7%)	0	4 (14%)	
cN3	0	0	0	
cNX	1 (2%)	1 (4%)	0	
Tumor grading				
G1	0	0	0	0.023
G2	24 (44%)	15 (60%)	9 (31%)	
G3	29 (54%)	9 (36%)	20 (69%)	
Missing	1 (2%)	1 (4%)	0	
Ki-67 score	40 (25–70)	40 (25–70)	40 (30–70)	0.676
ER- and PR-status				
Both negative	11 (20%)	5 (20%)	6 (21%)	0.950
One or both positive	43 (80%)	20 (80%)	23 (79%)	
HER-2 status				
Positive	17 (31%)	9 (36%)	8 (28%)	0.511
Negative	37 (69%)	16 (64%)	21 (72%)	
Breast cancer subtype				
HER-2 neg., HR pos	26 (48%)	11 (44%)	15 (52%)	0.790
HER-2 neg., HR neg	11 (20%)	5 (20%)	6 (21%)	
HER-2 pos., HR pos	17 (32%)	9 (36%)	8 (28%)	
HER-2 pos., HR neg	0	0	0	
ECOG				
0	51 (94%)	23 (92%)	28 (97%)	0.471
1	3 (6%)	2 (8%)	1 (3%)	
2–4	0	0	0	
BMI	24 (22–28)	23 (22–28)	25 (23–29)	0.139
Concomittant disease				
No	13 (24%)	6 (24%)	7 (24%)	0.991
Yes	41 (76%)	19 (76%)	22 (76%)	
Vascular	10 (19%)	4 (16%)	6 (21%)	
Endocrine	8 (15%)	3 (12%)	5 (17%)	
Cardiac	3 (6%)	2 (8%)	1 (3%)	
Psychiatric	3 (6%)	2 (8%)	1 (3%)	
MFI (Baseline)				
General Fatigue	9 (6–11)	10 (6–14)	9 (6–10)	0.396
FACT-B (Baseline)				
TOI	74 (63–81)	67 (61–80)	77 (67–82)	0.151

Standard neoadjuvant chemotherapy implies a three-weekly anthracycline administration, dose-dense a two-weekly administration

Data are *N* (%) or median (IQR), *P*-values compare the standard and the dose-dense subcohorts

Baseline characteristics were statistically different between the 29 patients receiving dose-dense chemotherapy and the 25 patients receiving standard chemotherapy in five parameters. Patients in the dose-dense chemotherapy group were more often premenopausal, had higher tumor grading and tended to be younger. Furthermore, the dose-dense chemotherapy group had less physical fatigue at baseline and a better FACT-B score in physical well-being (Table 1 and S1).

### Course of fatigue

During neoadjuvant chemotherapy, all MFI scales increased by more than two points after 25 weeks of treatment (all *P* < 0.001) (Table 2). Patients started chemotherapy with a mean general fatigue of 9.0, which increased by 3.4 points after 25 weeks of treatment. Physical fatigue and reduced activity increased the most, by 4.8 and 4.4 points, respectively. Reduced motivation and mental fatigue increased the least, by 2.4 and 2.1 points, respectively.

**Table 2:** Estimated mean baseline and change of score of the MFI and the FACT-B subscales after 25 weeks of neoadjuvant chemotherapy

Scale	Subscale	Baseline score (mean)	Score change from baseline after 25 weeks		
			Estimate	95% CI	
MFI	General fatigue	9.0	3.4^†††^	2.3	4.6
	Physical fatigue	8.3	4.8^†††^	3.7	6.0
	Reduced activity	8.0	4.4^†††^	3.1	5.7
	Reduced motivation	7.0	2.5^†††^	1.4	3.5
	Mental fatigue	8.2	2.1^†††^	1.0	3.2
FACT-B	FACT-B total score	111.7	− 9.5^††^	− 15.5	− 3.6
	Trial Outcome Index	71.0	− 9.8^†††^	− 14.4	− 5.1
	Physical well-being	23.7	− 4.1^†††^	− 6.0	− 2.2
	Social well-being	24.7	− 1.6^†^	− 3.0	− 0.2
	Emotional well-being	15.6	2.2^††^	0.8	3.7
	Functional well-being	17.0	− 2.9^††^	− 4.8	− 1.0
	Breast cancer subscale	29.4	− 2.1^†^	− 4.1	− 0.1

Statistical significance of the change from baseline: ^†^*p* < 0.05; ^††^*p* < 0.01; ^†††^*p* < 0.001

Analyses of treatment parameters showed only one statistically significant difference between the standard and the dose-dense chemotherapy group, where physical fatigue increased by 2.6 points more in the former than in the latter (Table 3). Comparing Epi ± Cyc and Pac ± Car cycles, the MFI scores always increased more during Epi ± Cyc cycles, but the difference between both groups did not become statistically significant in any dimension (Table S2).

**Table 3:** Estimated change of score of the MFI and the FACT-B subscales after 25 weeks of either standard or dose-dense neoadjuvant chemotherapy

Scale	Subscale	Score change from baseline after 25 weeks								
		Standard			Dose-Dense			Difference		
		Estimate	95% CI		Estimate	95% CI		Estimate	95% CI	
MFI	General fatigue	5.6^†††^	3.8	7.3	3.3^†††^	1.6	4.9	2.3	0.0	4.7
	Physical fatigue	6.1^†††^	4.4	7.8	3.5^†††^	1.9	5.1	2.6^†^	0.3	4.9
	Reduced activity	5.1^†††^	3.3	7.0	3.7^†††^	1.9	5.5	1.4	− 1.2	4.0
	Reduced motivation	2.8^†††^	1.3	4.4	0.9	− 0.6	2.3	2.0	− 0.1	4.1
	Mental fatigue	2.2^††^	0.6	3.9	2.0^†^	0.4	3.5	0.3	− 2.0	2.6
FACT-B	FACT-B total score	− 16.6^†††^	− 24.9	− 8.2	− 2.5	− 11.1	6.1	− 14.1^†^	− 26.1	− 2.0
	Trial Outcome Index	− 16.1^†††^	− 22.6	− 9.6	− 3.5	− 10.2	3.2	− 12.6^††^	− 21.9	− 3.2
	Physical well-being	− 6.6^†††^	− 9.3	− 3.9	− 1.6	− 4.3	1.1	− 4.9^†^	− 8.8	− 1.1
	Social well-being	− 2.1^†^	− 4.1	− 0.2	− 1.1	− 3.0	0.9	− 1.1	− 3.8	1.7
	Emotional well-being	2.0	− 0.1	4.1	2.5^†^	0.4	4.5	− 0.5	− 3.5	2.5
	Functional well-being	− 5.3^†††^	− 8.0	− 2.6	− 0.4	− 3.1	2.3	− 4.9^†^	− 8.7	− 1.1
	Breast cancer subscale	− 3.7^††^	− 6.5	− 1.0	− 0.5	− 3.4	2.3	− 3.2	− 7.2	0.8

Statistical significance of the change from baseline and of the difference between standard and dose-dense subcohorts: ^†^*p* < 0.05; ^††^*p* < 0.01; ^†††^*p* < 0.001

Further sensitivity analyses of the statistical model showed that the lower the general fatigue score was at baseline, the higher its average increase after 25 weeks of treatment (Table S3). For patients with the lowest general fatigue at baseline, 4.0 points, the model estimated an average score increase of 8.3 points. On the opposite, with the highest observed general fatigue at baseline, 16.0 points, the score was estimated to decrease by 3.3 points. In the present data set, general fatigue increased if baseline scores were below 12.0 and decreased if baseline scores were above. Multivariate analyses of further parameters did not indicate any statistically significant influence of BMI, UICC stage or age on the MFI score development (Table S4).

### Course of quality of life

During neoadjuvant chemotherapy, the TOI scale, and all the subscales it summarizes, decreased in a statistically significant manner after 25 weeks of treatment (Table 2). Patients started chemotherapy with a mean TOI score of 71.0, which decreased by 9.8 points. The subscales that the TOI summarizes, i.e., the physical, the functional well-being and the breast cancer subscales, decreased the most, by 4.1, 2.9 and 2.1 points, respectively. Emotional well-being was the only dimension to increase over the entire therapy, by 2.2 points.

Analyses of treatment parameters showed that physical well-being decreased more during standard chemotherapy (− 4.9 points) and during Epi ± Cyc cycles (− 2.2 points) than during dose-dense chemotherapy and Pac ± Car cycles, respectively (Table 3 and Table S5). Other FACT-B scales also decreased more in the standard than in the dose-dense chemotherapy group, i.e., the functional well-being, the TOI and the FACT-B total scale, by − 4.9, − 12.6 and − 14.1 points, respectively. During Epi ± Cyc and Pac ± Car cycles, all FACT-B scales decreased more during Epi ± Cyc cycles, but the difference in score decrease only became statistically significant for physical well-being (Table S5). In addition, the multivariate analysis did not indicate any statistically significant influence of BMI, UICC stage or age on the FACT-B total score after 25 weeks of treatment (Table S6).

### Incidence, severity and rate of adverse events

Analysis of adverse events considered only patients receiving Epi ± Cyc and Pac ± Car and distinguished between new onset and ongoing adverse events. Forty patients received Epi ± Cyc as first and Pac ± Car as second drug combination (Table 4). Ten patients received Pac ± Car as first drug combination and nine Epi ± Cyc as second combination.

**Table 4:** Incidence of the most documented adverse events (No. 1–7) and of the adverse events with grade 3 according to CTCAE (No. 8–12)

No.	Adverse event	New onset /ongoing	Number of patients affected during Epi ± Cyc cycles New onset: *N* = 49; Ongoing: *N* = 9			Number of patients affected during Pac ± Car cycles New onset: *N* = 50; Ongoing: *N* = 40		
			Grade 1	Grade 2	Grade 3	Grade 1	Grade 2	Grade 3
1	Alopecia	New onset	12 (24%)	23 (47%)	0	5 (10%)	7 (14%)	0
		Ongoing	3 (33%)	4 (44%)	0	6 (15%)	23 (58%)	0
2	Nausea	New onset	21 (43%)	7 (14%)	0	4 (8%)	1 (2%)	0
		Ongoing	1 (11%)	1 (11%)	0	8 (20%)	2 (5%)	0
3	Pain symptoms	New onset	20 (41%)	2 (4%)	0	19 (38%)	2 (4%)	0
		Ongoing	1 (11%)	0	0	13 (33%)	2 (5%)	0
4	Peripheral sensory neuropathy	New onset	4 (8%)	0	0	19 (38%)	3 (6%)	0
		Ongoing	3 (33%)	0	0	3 (8%)	0	0
5	Fatigue	New onset	11 (23%)	0	0	9 (18%)	1 (2%)	0
		Ongoing	1 (11%)	0	0	7 (18%)	0	0
6	Diarrhoea	New onset	10 (20%)	0	0	9 (18%)	2 (4%)	0
		Ongoing	0	0	0	4 (10%)	0	0
7	Anemia	New onset	6 (12%)	3 (6%)	0	2 (4%)	2 (4%)	0
		Ongoing	0	1 (11%)	0	5 (13%)	2 (5%)	0
8	Pyrexia	New onset	4 (8%)	0	1 (2%)	2 (4%)	0	1 (2%)
		Ongoing	0	0	0	0	0	0
9	Febrile neutropenia	New onset	1 (2%)	1 (2%)	2 (4%)	0	0	0
		Ongoing	0	0	0	0	0	0
10	Infection	New onset	4 (8%)	0	0	3 (6%)	0	1 (2%)
		Ongoing	0	0	0	0	0	0
11	Erythema	New onset	0	0	0	1 (2%)	0	1 (2%)
		Ongoing	0	0	0	0	0	0
12	Pneumonia	New onset	1 (2%)	1 (2%)	0	0	0	1 (2%)
		Ongoing	0	0	0	0	0	0

The adverse events’ highest documented CTCAE grade is shown. Adverse events defined as ‘new onset’ were first documented during the indicated drug combination (Epi ± Cyc or Pac ± Car), ‘ongoing’ adverse events were first documented during the previous drug combination. No statistically significant differences were found between Epi ± Cyc or Pac ± Car groups regarding the incidence of adverse events of grade ≥ 2

Most adverse events were grade 1–2, while only six patients experienced seven adverse events of grade 3 (Table 4). Seven MedDRA preferred term classes of adverse events, all of grade 1–2, occurred in more than 20% of the patients, i.e., alopecia, nausea, pain symptoms, peripheral sensory neuropathy, fatigue, diarrhoea and anemia. Adverse events of grade 3 included pyrexia, febrile neutropenia, infection, erythema and pneumonia. No statistically significant differences were observed between Epi ± Cyc or Pac ± Car groups regarding the incidence of adverse events of grade ≥ 2. Two patients terminated chemotherapy early due to grade 3 adverse events, one patient during Epi ± Cyc cycles, one during Pac ± Car cycles (Table S7).

The risk of experiencing adverse events in general was not significantly different between Epi ± Cyc and Pac ± Car cycles, neither for new onset adverse events during first (P = 0.173) or second sequences (*P* = 0.850), nor for ongoing adverse events (*P* = 0.229) (Table 6). Patients, however, experienced significantly more new adverse events during the first chemotherapy sequence than during the second, independent of the administered regimen (Epi ± Cyc 1st vs. 2nd sequence: HR = 0.63 vs. HR = 0.29; RR = 2.20; Pac ± Car 1st vs. 2nd: HR = 0.47 vs. HR = 0.30; RR = 1.56) (Tables 5 and 6). Moreover, considering the two alternative sequence arrangements, in the arrangement Epi ± Cyc followed by Pac ± Car (Epi ± Cyc | Pac ± Car) patients had a higher risk of new onset AEs in each of the two sequences than in the alternative arrangement (Epi ± Cyc | Pac ± Car: 0.63 | 0.30; Pac ± Car | Epi ± Cyc: 0.47 | 0.29).

**Table 5:** Number of adverse events documented during Epi ± Cyc and Pac ± Car cycles, with the according hazard rate of adverse events (total number of adverse events per week and per patient)

New onset/Ongoing	Drug combination	Sequence no.	No. of adverse events	No. of patients	Average no. of weeks	Hazard rate of adverse events (adverse events/ patient/ week)		
						Mean	95% CI	
New Onset	Epi ± Cyc	1	257	40	9.6	0.63	0.36	1.09
		2	19	9	11.2	0.29	0.13	0.61
		1 + 2	276	49	9.9	0.42	0.23	0.77
	Pac ± Car	1	56	10	14.7	0.47	0.25	0.89
		2	187	40	14.1	0.30	0.17	0.53
		1 + 2	243	50	14.2	0.38	0.22	0.66
Ongoing	Epi ± Cyc	2	19	9	12.1	0.17	0.11	0.28
	Pac ± Car	2	132	40	14.1	0.23	0.20	0.28

**Table 6:** Risk ratio of hazard rates during drug combinations either given first (sequence no. 1) or second (sequence no. 2)

New onset/Ongoing	Comparator 1		Comparator 2		Relative Risk (AE/week)	95% CI		*P*-value
	Drug combination	Sequence no.	Drug combination	Sequence no.				
New Onset	Epi ± Cyc	1	Epi ± Cyc	2	2.20	1.22	3.98	0.010
	Epi ± Cyc	1	Pac ± Car	1	1.33	0.88	2.02	0.173
	Epi ± Cyc	2	Pac ± Car	2	0.94	0.52	1.72	0.850
	Pac ± Car	1	Pac ± Car	2	1.56	1.02	2.39	0.041
Ongoing	Epi ± Cyc	2	Pac ± Car	2	0.74	0.45	1.21	0.229

## Discussion

This cohort study is to our knowledge the largest real world, multidimensional examination of CRF and HRQoL in early breast cancer patients undergoing neoadjuvant chemotherapy. Fifty-four patients’ answers of the MFI and the FACT-B were used to measure CRF and HRQoL repeatedly over the course of chemotherapy.

On average, early breast cancer patients experienced a clinically significant worsening of general fatigue and of the FACT-B-TOI during neoadjuvant chemotherapy. General fatigue and TOI deteriorated by 3.4 and 9.8 points, respectively, thereby reaching statistical and clinical significance [20, 21]. These results are similar to other findings collected during neoadjuvant chemotherapy of early breast cancer, where the MFI general fatigue scale increased by 2.2 points from a baseline of 11.3 [13] and the TOI decreased by 16 points from a baseline of 76 [15].

Comparisons with the literature show the following: first, our results suggest that CRF during chemotherapy remains an unmet clinical need. While early breast cancer patients start neoadjuvant chemotherapy with an average fatigue comparable to that of the general population [6], they end this treatment on average with a critical level of CRF [22].

Second, physical dimensions of CRF and HRQoL worsen more than other dimensions during neoadjuvant chemotherapy. Our study confirmed previous results on CRF in the adjuvant and neoadjuvant setting, where physical fatigue had worsened, while mental fatigue and reduced motivation had not changed [11, 13, 14]. Similarly, for HRQoL, our study confirmed previous results where physical well-being worsened during neoadjuvant chemotherapy, while emotional well-being improved [12].

Third, our results suggest that the baseline fatigue score is a better prognostic factor for the course of CRF than age. This new hypothesis does not contradict previous findings. Hinz and colleagues showed that the general population had less fatigue in younger and more fatigue in older age groups (18–40 years vs. above 61) [6]. In the cancer population, however, all age groups had a similarly high fatigue score, thereby suggesting that cancer led to a stronger fatigue increase in younger patients. While our study did not identify any correlation between age and the worsening of fatigue, we identified a correlation with the baseline fatigue score: the lower the fatigue at baseline, the stronger its worsening during chemotherapy. This more general correlation can also be applied to the data of Hinz and colleagues. Further research is needed to confirm this observation.

Fourth, we can confirm that neither BMI at baseline nor cancer stage I–III has an impact on CRF. Reinertsen and colleagues did not observe differences in CRF change between obese (BMI ≥ 30) and non-obese (BMI < 30) patients [10]. These 84 patients (17 obese) received a treatment similar to patients in the present study: four three-weekly cycles of fluoruracil, epirubicin and cyclophosphamide followed by twelve weekly cycles of paclitaxel or four three-weekly cycles of docetaxel. While BMI has been reported to be a predictor for long-term CRF in cancer survivors [23], it does not seem to play a role during chemotherapy itself. Regarding the impact of the cancer stage on CRF, we could confirm the results of Hinz and colleagues, who found no difference in CRF-levels in cancer patients of stages I–III [6].

Fifth, the impact of the density of anthracycline cycles (dose-dense vs. standard) on CRF and HRQoL remains unclear. Our results contradict earlier published data [7]. We observed a tendency for a stronger worsening of CRF and HRQoL during standard neoadjuvant chemotherapy, which becomes significant in the physical dimensions of both endpoints. Brandberg and colleagues, however, observed that patients under standard adjuvant chemotherapy had better HRQoL and less CRF than those under dose-dense chemotherapy did [8]. Brandberg’s results have more weight than ours, as they originate from a randomized controlled trial. Although our two groups do not differ for most demographic and anamnestic parameters, we cannot exclude differences in undocumented parameters. Particularly, both groups started neoadjuvant chemotherapy from statistically different scores in the physical dimensions, the standard chemotherapy group having worse baseline scores than the dose-dense group. This suggests that dose-dense chemotherapy was preferably administered to patients with a better health status at start of therapy, who might be able to tolerate the expected higher toxicity of this therapy.

Sixth, while CRF, HRQoL and adverse events are not statistically different during anthracycline and taxane cycles, we observed a trend that these parameters become worse under Epi ± Cyc. Zaheed and colleagues reported results of one study in the adjuvant setting that did not show any statistical difference in HRQoL regarding the sequence arrangement of taxanes and anthracyclines in breast cancer patients [9]. Like us, they could find some indication that a taxane first arrangement may result in a slightly reduced overall risk of adverse events, e.g. for grade 3/4 neutropenia. Also, the hypothesis that CRF, HRQoL and adverse events are worse under anthracyclines should not be ruled out yet, as Zaheed and colleagues concede that their conclusions are based on few studies and require further research.

The impact of the risk factors shown above requires further research, as the synergies of multiple risk factors are not fully understood. Comorbidities known to influence CRF were balanced for the evaluation of certain risk factors (e.g. concomitant diseases at baseline for dose-dense and standard groups; adverse events for Epi ± Cyc and Pac ± Car groups). This balance, however, cannot be guaranteed for all comparisons. Furthermore, other factors influencing CRF such as social parameters (e.g. marital status, income level) and certain inflammatory markers [23] need to be documented in future studies to give a complete picture.

## Conclusion

Our results suggest that further actions to reduce CRF and improve HRQoL during neoadjuvant chemotherapy of early breast cancer patients are needed. Physical dimensions of CRF and HRQoL are particularly impacted by neoadjuvant chemotherapy. Future research should investigate the impact of different chemotherapy sequences and densities on CRF and HRQoL.

### Supplementary Information

Below is the link to the electronic supplementary material.

Supplementary file1 (DOCX 38 kb)

## Data Availability

The authors state that they have full control of all primary data and agree to allow the journal to review this data if requested.

## References

[1] Hyder T, Bhattacharya S, Gade K, Nasrazadani A, Brufsky A (2021). Approaching neoadjuvant therapy in the management of early-stage breast cancer. Breast Cancer (Dove Med Press).

[2] Ortmann O, Blohmer J, Sibert N, Brucker S, Janni W, Wöckel A (2022). Current clinical practice and outcome of neoadjuvant chemotherapy for early breast cancer: analysis of individual data from 94,638 patients treated in 55 breast cancer centers. J Cancer Res Clin Oncol.

[3] Riedel F, Hoffmann A, Moderow M, Heublein S, Deutsch T, Golatta M (2020). Time trends of neoadjuvant chemotherapy for early breast cancer. Int J Cancer.

[4] Ditsch N, Untch M, Thill M, Müller V, Janni W, Albert U (2019). AGO Recommendations for the diagnosis and treatment of patients with early breast cancer: update 2019. Breast Care (Basel).

[5] Leitlinienprogramm Onkologie (Deutsche Krebsgesellschaft, Deutsche Krebshilfe, AWMF) S3 Leitlinie Früherkennung, Diagnose, Therapie und Nachsorge des Mammakarzinoms, Version 4.3. 2021; https://www.leitlinienprogramm-onkologie.de/leitlinien/mammakarzinom. Accessed 23 Sep 2023.

[6] Hinz A, Weis J, Brähler E, Härter M, Geue K, Ernst J (2020). Fatigue in cancer patients: comparison with the general population and prognostic factors. Support Care Cancer.

[7] Denlinger C, Ligibel J, Are M, Baker K, Demark-Wahnefried W, Friedman D (2014). Survivorship: fatigue, version 1.2014. J Natl Compr Canc Netw.

[8] Brandberg Y, Johansson H, Hellström M, Gnant M, Möbus V, Greil R (2020). Long-term (up to 16 months) health-related quality of life after adjuvant tailored dose-dense chemotherapy vs. standard three-weekly chemotherapy in women with high-risk early breast cancer. Breast Cancer Res Treat.

[9] Zaheed M, Wilcken N, Willson M, O'Connell D, Goodwin A (2019). Sequencing of anthracyclines and taxanes in neoadjuvant and adjuvant therapy for early breast cancer. Cochrane Database Syst Rev.

[10] Reinertsen K, Engebraaten O, Loge J, Cvancarova M, Naume B, Wist E (2017). Fatigue during and after breast cancer therapy-a prospective study. J Pain Symptom Manag.

[11] de Jong N, Candel M, Schouten H, Abu-Saad H, Courtens A (2004). Prevalence and course of fatigue in breast cancer patients receiving adjuvant chemotherapy. Ann Oncol.

[12] Hornsby W, Douglas P, West M, Kenjale A, Lane A, Schwitzer E (2014). Safety and efficacy of aerobic training in operable breast cancer patients receiving neoadjuvant chemotherapy: a phase II randomized trial. Acta Oncol.

[13] Jong M, Boers I, Schouten van der Velden A, Meij S, Göker E, Timmer-Bonte A (2018). A randomized study of yoga for fatigue and quality of life in women with breast cancer undergoing (Neo) adjuvant chemotherapy. J Altern Complement Med.

[14] de Jong N, Candel M, Schouten H, Abu-Saad H, Courtens A (2005). Course of mental fatigue and motivation in breast cancer patients receiving adjuvant chemotherapy. Ann Oncol.

[15] Okuyama H, Nakamura S, Akashi-Tanaka S, Sawada T, Kuwayama T, Handa S (2018). QOL evaluation of nab-paclitaxel and docetaxel for early breast cancer. Eur J Breast Health.

[16] Zhao Y, Chen L, Zheng X, Shi Y (2022). Quality of life in patients with breast cancer with neoadjuvant chemotherapy: a systematic review. BMJ Open.

[17] Person H, Guillemin F, Conroy T, Velten M, Rotonda C (2020). Factors of the evolution of fatigue dimensions in patients with breast cancer during the 2 years after surgery. Int J Cancer.

[18] Smets EM, Garssen B, Cull A, de Haes JC (1996). Application of the multidimensional fatigue inventory (MFI-20) in cancer patients receiving radiotherapy. Br J Cancer.

[19] Brady MJ, Cella DF, Mo F, Bonomi AE, Tulsky DS, Lloyd SR (1997). Reliability and validity of the Functional Assessment of Cancer Therapy-Breast quality-of-life instrument. J Clin Oncol.

[20] Eton D, Cella D, Yost K, Yount S, Peterman A, Neuberg D (2004). A combination of distribution- and anchor-based approaches determined minimally important differences (MIDs) for four endpoints in a breast cancer scale. J Clin Epidemiol.

[21] Purcell A, Fleming J, Bennett S, Burmeister B, Haines T (2010). Determining the minimal clinically important difference criteria for the Multidimensional Fatigue Inventory in a radiotherapy population. Support Care Cancer.

[22] Schwarz R, Krauss O, Hinz A (2003). Fatigue in the general population. Onkologie.

[23] Bower J, Wiley J, Petersen L, Irwin M, Cole S, Ganz P (2018). Fatigue after breast cancer treatment: biobehavioral predictors of fatigue trajectories. Health Psychol.

