# Supplementary material for: Fatigue and quality of life during neoadjuvant chemotherapy of early breast cancer: a prospective multicenter cohort study
Source: Breast Cancer. 2023 Nov 15;31(1):124–34. doi: 10.1007/s12282-023-01520-y (PMC10764505; doi:10.1007/s12282-023-01520-y)
Supplement: Supplementary file 1 — Supplementary file1 (DOCX 38 kb) [file 12282_2023_1520_MOESM1_ESM.docx]

**Supplementary data to**

**“Fatigue and Quality of Life During Neoadjuvant Chemotherapy of Early Breast Cancer: A Prospective Multicenter Cohort Study”**

Florian Pelzer ^1,2^, Wilfried Tröger ^2^, Marcus Reif ^3^, Susanne Schönberg ^3^, David D. Martin^1,4^,

Cornelia Müller ^5^, Isabell Utz-Billing ^6^, Thorsten Kühn ^7,8^, Stephan Baumgartner^1,2,3^, Marion Kiechle ^9^ and Daniela Paepke ^9,10^

^1^ *Institute for Integrative Medicine, Witten/Herdecke University, Witten, Germany*

^2^ *Society for Cancer Research, Arlesheim, Switzerland*

^3^ *Gesellschaft für klinische Forschung e.V., Berlin, Germany*

^4^ *Tübingen University Children’s Hospital, Tübingen, Germany*

^5^ *Brandenburgisches Brustzentum, Universitätsklinikum Brandenburg an der Havel, Brandenburg an*

*der Havel, Germany*

^6^ *Brustzentrum, Park-Klinik Weissensee, Berlin, Germany*

^7^ *Klinik für Frauenheilkunde und Geburtshilfe, Klinikum Esslingen, Esslingen, Germany*

^8^ *Filderklinik, Filderstadt, Germany*

^9^ *Department of Obstetrics and Gynecology, Klinikum Rechts der Isar, Technical University of Munich,*

*Munich, Germany.*

^10^ *Spital Zollikerberg, Zurich, Switzerland*

Table S1. Baseline values for the MFI-dimensions and FACT-B scores and subscales of the total patient cohort and of the subcohorts receiving standard or dose-dense neoadjuvant chemotherapy. Standard neoadjuvant chemotherapy implies a three-weekly anthracycline administration, dose-dense a two-weekly administration.

|  | | **Total** | **Standard** | **Dose-dense** | **P-value** |
| --- | --- | --- | --- | --- | --- |
| **MFI** | **General fatigue** | 9 (6–11) | 10 (6–14) | 9 (6–10) | 0.396 |
|  | **Physical fatigue** | 8 (5–10) | 10 (7–11) | 7 (5–9) | 0.041 |
|  | **Reduced activity** | 7 (6–10) | 9 (6–11) | 6 (6–9) | 0.148 |
|  | **Reduced motivation** | 7 (5–8) | 6 (5–8) | 8 (5–8) | 0.335 |
|  | **Mental fatigue** | 8 (5–10) | 9 (5–10) | 8 (4–10) | 0.404 |
| **FACT-B** | **FACT-B total score** | 116 (99–126) | 107 (96–123) | 119 (105–127) | 0.182 |
|  | **Trial Outcome Index** | 74 (63–81) | 67 (61–80) | 77 (67–82) | 0.151 |
|  | **Physical well-being** | 26 (22–27) | 23 (19–27) | 26 (25–28) | 0.027 |
|  | **Social well-being** | 26 (23–27) | 26 (22–27) | 26 (24–28) | 0.657 |
|  | **Emotional well-being** | 17 (12–20) | 15 (9–20) | 18 (15–20) | 0.140 |
|  | **Functional well-being** | 18 (13–22) | 14 (11–22) | 20 (15–23) | 0.091 |
|  | **Breast cancer subscale** | 30 (26–33) | 29 (25–34) | 30 (27–33) | 0.633 |

Data are median (IQR), P-values compare the standard and the dose dense subcohorts.

Table S2. Influence of two drug combinations (Epi±Cyc and Pac±Car) on the estimated change of score for the five MFI-dimensions

| **MFI Dimension** | **Score change from baseline** | | | | | | | | |
| --- | --- | --- | --- | --- | --- | --- | --- | --- | --- |
|  | **After 6 weeks Epi±Cyc** | | | **After 11 weeks Pac±Car** | | | **Difference** | | |
|  | **Estimate** | **95% CI** | | **Estimate** | **95% CI** | | **Estimate** | **95% CI** | |
| **General fatigue** | 2.0^†††^ | 1.1 | 3.0 | 1.5^†^ | 0.3 | 2.6 | 0.6 | −1.0 | 2.2 |
| **Physical fatigue** | 2.1^†††^ | 1.2 | 3.0 | 1.3^†^ | 0.2 | 2.5 | 0.8 | −0.8 | 2.4 |
| **Reduced activity** | 1.9^†††^ | 1.0 | 2.8 | 1.1 | −0.1 | 2.3 | 0.8 | −0.8 | 2.4 |
| **Reduced motivation** | 0.7^†^ | 0.0 | 1.4 | -0.2 | −1.2 | 0.9 | 0.9 | −0.5 | 2.3 |
| **Mental fatigue** | 1.0^†^ | 0.2 | 1.8 | 0.0 | −1.1 | 1.1 | 1.0 | −0.5 | 2.5 |

Statistical significance of the change from baseline and of the difference between Epi±Cyc and Pac±Car subcohorts: †: p < 0.05;

††: p < 0.01; †††: p < 0.001

Table S3. Influence of the baseline scores on the estimated change of score for the General Fatigue dimension after 25 weeks of neoadjuvant chemotherapy. The other dimensions behave in a similar manner, i.e. the higher the baseline score, the lower the score increase. Only the range of baseline scores observed in this study (4.0 – 16.0) are shown.

| **MFI Dimension** | **Baseline Score** | **Score change after 25 weeks** | | | **P-value** |
| --- | --- | --- | --- | --- | --- |
|  |  | **Estimate** | **95% CI** | |  |
| **General**  **fatigue** | 4.0 | 8.3 | 6.2 | 10.3 | <.001 |
| **General**  **fatigue** | 6.0 | 6.3 | 4.8 | 7.9 | <.001 |
| **General**  **fatigue** | 8.0 | 4.4 | 3.2 | 5.6 | <.001 |
| **General**  **fatigue** | 10.0 | 2.5 | 1.3 | 3.7 | <.001 |
| **General**  **fatigue** | 12.0 | 0.6 | −1.0 | 2.1 | 0.470 |
| **General**  **fatigue** | 14.0 | −1.4 | −3.4 | 0.7 | 0.187 |
| **General**  **fatigue** | 16.0 | −3.3 | −5.9 | -0.7 | 0.014 |

Table S4. Multivariate confounder analysis on the influence of BMI, UICC stage and age on the change of average MFI-score after 25 weeks of neoadjuvant chemotherapy

| **Confounder** | **Type of analysis** | **Estimate** | **95% CI** | | **P-value** |
| --- | --- | --- | --- | --- | --- |
| **BMI** | Difference of BMI (≥25 minus <25) for MFI-score at baseline | −0.05 | −1.6 | 1.50 | 0.952 |
|  | Difference of BMI (≥25 minus <25) for MFI-score after 25 wks. | 0.85 | −1.05 | 2.76 | 0.376 |
| **UICC stage** | Influence of UICC (per stage) on MFI-score after 25 wks. | −0.57 | −1.44 | 0.31 | 0.200 |
| **Age** | Influence of age (per year) on MFI-score after 25 wks. | −0.02 | −0.12 | 0.09 | 0.764 |

Table S5. Influence of two drug combinations (Epi±Cyc and Pac±Car) on the estimated change of FACT-B summary scores and subscale score

| **FACT-B**  **scores and subscales** | **Score change from baseline** | | | | | | | | |
| --- | --- | --- | --- | --- | --- | --- | --- | --- | --- |
|  | **After 6 weeks Epi±Cyc** | | | **After 11 weeks Pac±Car** | | | **Difference** | | |
|  | **Estimate** | **95% CI** | | **Estimate** | **95% CI** | | **Estimate** | **95% CI** | |
| **FACT-B**  **total score** | −3.1 | −6.2 | 0.1 | −1.8 | −6.4 | 2.8 | −1.3 | −7.6 | 5.0 |
| **Trial Outcome**  **Index** | −4.7^†††^ | −7.2 | −2.2 | −1.3 | −5.0 | 2.5 | −3.4 | −8.6 | 1.7 |
| **Physical**  **well-being** | −2.4^†††^ | −3.5 | −1.3 | −0.2 | −1.8 | 1.4 | −2.2^†^ | −4.4 | 0.0 |
| **Social**  **well-being** | −0.3 | −1.1 | 0.4 | −0.5 | −1.5 | 0.5 | 0.2 | −1.2 | 1.6 |
| **Emotional**  **well-being** | 1.3^††^ | 0.5 | 2.1 | 0.6 | −0.5 | 1.8 | 0.7 | −0.9 | 2.2 |
| **Functional**  **well-being** | −1.8^†††^ | −2.8 | −0.8 | 0.0 | −1.5 | 1.5 | −1.8 | −3.9 | 0.3 |
| **Breast cancer subscale** | −0.7 | −1.7 | 0.3 | −0.7 | −2.2 | 0.8 | 0 | −2.1 | 2.0 |

Statistical significance of the change from baseline and of the difference between Epi±Cyc and Pac±Car subcohorts: †: p < 0.05;

††: p < 0.01; †††: p < 0.001

Table S6. Multivariate confounder analysis on the influence of BMI, UICC stage and age on the change of the FACT-B total score after 25 weeks of neoadjuvant chemotherapy

| **Confounder** | **Type of analysis** | **Estimate** | **95% CI** | | **P-value** |
| --- | --- | --- | --- | --- | --- |
| **BMI** | Difference of BMI (≥25 minus <25) for FACT-B total score at baseline | 0.97 | −6.10 | 8.03 | 0.787 |
|  | Difference of BMI (≥25 minus <25) for FACT-B total score after 25 wks. | −2.21 | −11.21 | 6.80 | 0.629 |
| **UICC stage** | Influence of UICC stage (1 unit) on FACT-B total score after 25 wks. | 0.20 | −4.28 | 4.69 | 0.929 |
| **Age** | Influence of age (1 unit) on FACT-B total score after 25 wks. | −0.11 | −0.66 | 0.45 | 0.71 |

Table S7. Patient list with reasons for premature chemotherapy termination. Patients DE02–08, 02–13, 03–02, 04–09 were included in the analysis until their time of drop-out, which ranged from 14 to 21 weeks after start of chemotherapy. They were on average 5 years younger, premenopausal (except one), with ECOG 0, nearly no current diseases, symptoms or medications, yet higher KI-67 values; all were planned for dose dense regimens. They started with less fatigue and higher quality of life scores in all dimensions of MFI and FACT-B, respectively.

| **Pat. No.** | **Reason for premature termination** | **MedDRA- Lowest Level Term** | **AE Severity** | **AE Grade** | **AE Cause** | **AE Action** |
| --- | --- | --- | --- | --- | --- | --- |
| **1.** | Lost to follow-up directly after qualifying examination |  |  |  |  |  |
| **2.** | Concomitant disease  (high GOT, GPT, GGT) |  |  |  |  |  |
| **3.** | Adverse event | Erythema | severe | 3 | paclitaxel | use discontinued permanently / termination of chemotherapy |
| **4.** | Adverse event | Pancytopenia | severe | 3 | cyclophos-phamide alone | use discontinued permanently / termination of chemotherapy |
| **5.** | Adverse event | Anaemia | moderate | 2 | epirubicin | use discontinued permanently / termination of chemotherapy |
|  |  | Neutropenic fever | moderate | 3 | epirubicin |  |
|  |  | Pneumonia | moderate | 3 | epirubicin |  |
